# Quantitative Analysis of Total Amino Acid in Barley Leaves under Herbicide Stress Using Spectroscopic Technology and Chemometrics

**DOI:** 10.3390/s121013393

**Published:** 2012-10-01

**Authors:** Yidan Bao, Wenwen Kong, Yong He, Fei Liu, Tian Tian, Weijun Zhou

**Affiliations:** 1 College of Biosystems Engineering and Food Science, Zhejiang University, Hangzhou 310058, China; E-Mails: ydbao@zju.edu.cn (Y.B.); zjukww@163.com (W.K.); yhe@zju.edu.cn (Y.H.); 2 College of Agriculture and Biotechnology, Zhejiang University, Hangzhou 310058, China; E-Mails: woshi.xiaot@163.com (T.T.); wjzhou@zju.edu.cn (W.Z.); 3 Cyrus Tang Center for Sensor Materials and Applications, Zhejiang University, Hangzhou 310058, China

**Keywords:** visible and near infrared spectroscopy, barley, total amino acid, variable selection, successive projections algorithm, least squares-support vector machine

## Abstract

Visible and near infrared (Vis/NIR) spectroscopy were employed for the fast and nondestructive estimation of the total amino acid (TAA) content in barley (*Hordeum vulgare* L.) leaves. The calibration set was composed of 50 samples; and the remaining 25 samples were used for the validation set. Seven different spectral preprocessing methods and six different calibration methods (linear and nonlinear) were applied for a comprehensive prediction performance comparison. Successive projections algorithm (SPA) and regression coefficients (RC) were applied to select effective wavelengths (EWs). The results indicated that the latent variables-least-squares-support vector machine (LV-LS-SVM) model achieved the optimal performance. The prediction results by LV-LS-SVM with raw spectra were achieved with a correlation coefficients (*r*) = 0.937 and root mean squares error of prediction (RMSEP) = 0.530. The overall results showed that the NIR spectroscopy could be used for determination of TAA content in barley leaves with an excellent prediction precision; and the results were also helpful for on-field monitoring of barley growing status under herbicide stress during different growth stages.

## 1. Introduction

Barley is one of the earliest cultivated cereal grains in the World, which is attracting renewed interest for its use in food and as a bioethanol feedstock [1]. It is a preferred grain for cultivation in many areas in the World due to its resistance to drought and ability to mature in climates with a short growing season [2]. Amino acid content is a very important physiological indicator which has a close relationship with the influence of environment stress during plant growing season. Recently, propyl 4-(2-(4,6-dimethoxypyrimidin-2-yloxy)benzylamino)benzoate (ZJ0273), a newly developed herbicide, has been applied to remove and control the weeds in barley fields. ZJ0273 is an ALS (acetolactate synthase)-inhibiting herbicide, which is considered to influence the formation of branch-chain amino acids (like aspartic acid, valine and proline) [3]. Hence, total amino acids (TAA) are basic physiological data and important parameters to understand the mechanism of herbicide effects on barley growth. The traditional amino acid detection method uses an automatic amino acid analyzer, which is laborious, time consuming, destructive and expensive. This method is not convenient for the fast and nondestructive detection of amino acids for field monitoring of plant growth information. Therefore, a rapid and practical method was necessary for the fast and accurate detection of amino acids.

Near infrared (NIR) spectroscopy is a common alternative analysis tool to traditional analytical methods. The NIR spectroscopy technique is rapid, and does not require labor-intensive sample processing, allowing for large-scale sampling [4]. It has developed rapidly in the past decades. In the agriculture field, NIR can be used to predict the neutral detergent fiber (NDF) and acid detergent fiber (ADF) of cereal residues from dryland cropping systems and is a useful tool to estimate residue decomposition potential [5]. Some researchers had shown the possibility of using NIR spectroscopy to analyze the β-glucan content in barley [6]. It is also possible to predict ergosterol content in whole barley samples using NIR [7]. The application of herbicides is an efficient and effective chemical weed control method to achieve optimal crop production [8], but herbicides also cause crop damage. Some physiological indicators are useful in evaluating the effect of herbicides [9]. This study was mainly focused on the feasibility of developing a rapid and effective method for the quantification of TAA in barley leaves using NIR spectroscopy to provide a new monitoring method for herbicide injury.

## 2. Material and Methods

### 2.1. Samples Preparation and Reflectance Measurements

Barley (*Hordeum vulgare* L.) used in our research was planted at the farm of Zhejiang University, Hangzhou (30°10′N, 120°12′E), China. The samples included 75 barley leaves, 50 for calibration and 25 for validation, and no single sample was used in both the calibration set and validation set at the same time. The calibration and validation set were randomly repeated several times in order to obtain a stable model. A new herbicide called ZJ0273 was applied during the seeding stage, the herbicide concentrations were 0, 50, 100, 500 and 1,000 mg/L, which are normally used for herbicide stress studies and practical field applications.

A Handheld FieldSpec spectrometer (Analytical Spectral Device, Boulder, CO, USA) was used within the 325–1,075 nm wavelength region for the reflectance spectral acquisition of all barley leaf samples. The resolution of this instrument is 1.5 nm. The reflectance mode was applied to obtain the spectra data of fresh barley leaves. The field-of-view (FOV) of the spectroradiometer is 25°. The distance between leaf sample and detector was 20 cm. Three replicate spectra were collected for each leaf sample, and the averaged spectrum obtained by averaging 30 scans per spectrum was used as the spectral data of each leaf sample. All spectra data were processed using the RS^3^ software for Windows (Analytical Spectral Devices, Boulder, CO, USA) with a Graphical User Interface. The software used in this study included ASD View Spec Pro, Unscrambler V9.8 (CAMO AS, Oslo, Norway) and MATLAB V7.0 (The Math Works, Natick, MA, USA). The pretreatment of leaf samples and the protocol for amino acid extraction was based on the Lisiewska method [10]. The content of TAA in barley leaves was determined using a Hitachi automatic amino acid analyzer L-8900 (Hitachi High-Technologies Corporation, Tokyo, Japan) under common detection conditions.

### 2.2. Data Pre-Treatment

Previous studies showed that pre-treatment of measured spectral data was an important strategy to improve prediction performance [11]. In order to achieve the optimal spectral preprocessing method to predict TAA in barley, several different spectral preprocessing methods were compared. Seven different preprocessing methods were applied, including Savitzky-Golay smoothing (SG), standard normal variate (SNV), multiplicative scatter correction (MSC), first-derivative (1-Der), second-derivative (2-Der), de-trending and direct orthogonal signal correction (DOSC). SG smoothing, SNV, and MSC can be used for de-noising, light scatter correction, and light pathlength correction [12,13]. Derivatives were applied to correct the baseline shift [11]. De-trending seeks to remove nonlinear trends in spectral data [14]. DOSC corrected the major variance sources such as temperature effects, time influences and instrumental differences in spectral data [15]. The performance was determined by the prediction results in the later calibration stage.

### 2.3. Multivariate Analysis

Partial least squares (PLS) is a chemometrics method which is widely applied in NIR spectroscopic techniques. It is a bilinear modeling method. Latent variables (LVs) were used as the direct inputs of the PLS models to develop a relationship between the spectral data and TAA in barley leaves. A full cross-validation procedure was performed to test the model development.

In order to compare different modeling methods, a least squares-support vector machine (LS-SVM) model was built in this study. It is a powerful calibration method to handle linear and nonlinear problems with a good statistical basis [16]. The details of LS-SVM can be found in the literature [17,18]. Herein, the PLS and LS-SVM methods were compared to obtain the optimal prediction model of TAA in barley. PLS model can develop a linear relationship between the spectra data and TAA in barley. However, there is some useful nonlinear information in the spectra data which could be helpful to improve prediction performance. Therefore, LS-SVM was investigated to develop a model using both linear and nonlinear information in spectra data. LS-SVM applies linear equations using support vectors instead of quadratic programming problems to reduce the complexity of the optimization processes, which has advantages for multivariate analysis.

There are several indicators relating to the quality of developed models. Correlation coefficients (*r*) and root mean squares error of prediction (RMSEP) were considered as the main evaluation standards in this study. An ideal model should have a high *r* value closing to 1 and a low RMSEP value.

### 2.4. Selection of the Effective Wavelengths (EWs)

Normally, the full spectra might contain hundreds of variables, therefore, removing uninformative variables was an effective strategy to get better prediction and simpler models. The research by Wold has shown that using optimum wavelengths might be equally or more efficient than using full wavelengths in multivariate analysis [19]. Regression coefficients (RC) analysis and successive projections algorithm (SPA) were employed to select the effective wavelengths in this study. Regression coefficient (RC) by performing PLS could be used as a way to select the effective wavelengths (EWs) [20]. The RC in the PLS model was used to calculate the response *Y*-variable from the *X*-variables. The coefficients gave an indication of which variables having the important impact on the response variables (*Y*). Large absolute values indicated the importance and the significance of the effect on the prediction of *Y*-variable. Successive projections algorithm (SPA) was a forward selection method which comprises three phases [21]. It starts with one wavelength, then incorporates a new one at each iteration, until a specified number of wavelengths is reached. With SPA, the informative variables with the least collinearity and redundancies could be selected. The selected EWs could be used as the direct input of the PLS and LS-SVM models.

### 2.5. Different Calibration Models

Different calibration methods were used for a better prediction of TAA in barley leaves under herbicide stress. Latent variables (LVs) were eigenvectors which were extracted during the building of the PLS model. Using LVs as the direct inputs of the PLS and LS-SVM models, the LV-PLS and LV-LS-SVM models were built. Based on the variables selected by SPA and RC, additional four different calibration models were developed, including SPA-PLS, RC-PLS, SPA-LS-SVM and RC-LS-SVM. The best model was achieved according to the prediction performance of the above mentioned calibration methods.

## 3. Results and Discussion

### 3.1. Results of Full-Spectral Models

**Figure 1.**
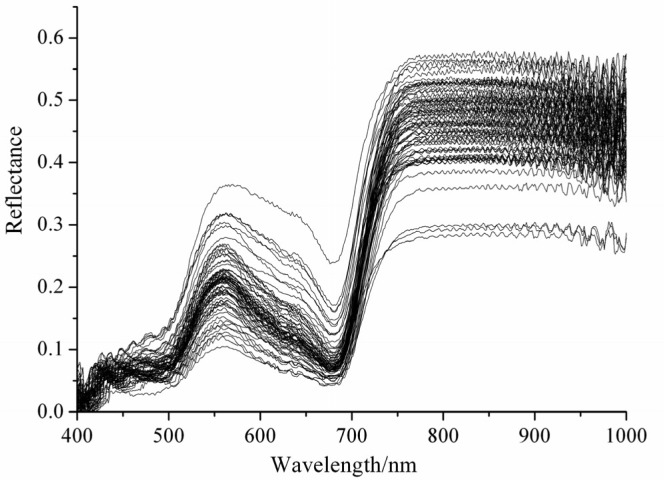
The original Vis/NIR reflectance spectra of barley leaves.

Figure 1 shows the original visible/near infrared reflectance spectra of 75 barley leaves. The trends of all samples with different herbicide concentrations were quite similar by visual inspection. There was a significant absorbance at around 680 nm caused by chlorophyll. The statistics of TAA in calibration and validation sets are shown in Table 1. Different PLS models were developed to find the optimal preprocessing methods. As the above-mentioned performance indicators, the correlation coefficients (*r*) and root mean squares error of prediction (RMSEP) were used to decide the quality of the calibration model.

Table 2 includes the prediction results of TAA in validation set by the PLS models with eight preprocessing methods. A full cross-validation was applied during PLS calibration. Different latent variables (LVs) were used in PLS models related with different spectral preprocessing methods. The optimal PLS model was achieved by Raw spectra with *r* = 0.879 and RMSEP = 0.751. The next best PLS model was the de-trending spectra based model. Raw and de-trending were considered the optimal preprocessing methods in this study and were used in the further analysis.

On the other hand, the prediction results by the PLS models with the full-spectrum data were not so good, with none of the correlation coefficients of these prediction results exceeding 0.9. A possible reason was that the full-spectrum models contained too many variables (601), and some uninformative ones inevitably weakened the prediction performance of the models. Hence, further improvement should be done to give a smaller number of variables which carry the useful information to build more sensitive models.

### 3.2. Selected EWs by SPA and RC

**Figure 2.**
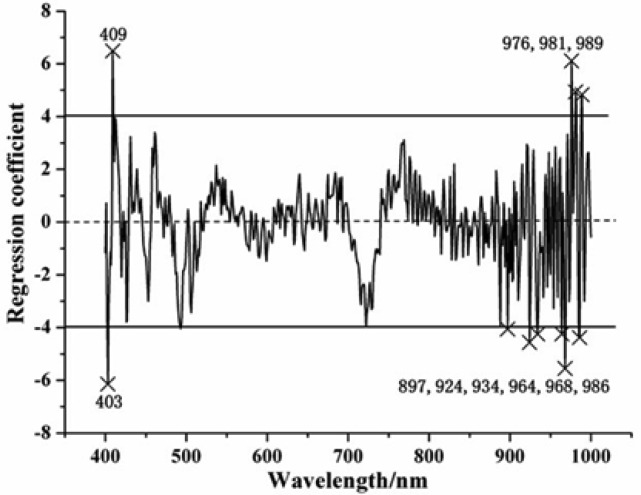
Selected effective wavelengths by regression coefficients.

As mentioned above, SPA and RC were used for the selection of EWs, and the optimal preprocessing methods were also taken into consideration. In SPA, the maximum number of selected variables was set as 30 according to experience and previous literature [22]. Based on experience and preliminary studies, there were two basic principles using RC: (1) the absolute RC value of selected EWs should be larger than certain threshold value, and (2) these selected EWs should at certain peaks and valleys of the regression coefficient curve plot [20]. Therefore, the threshold value was settled as ±4 in the RC analysis. The values of the regression coefficient which indicated the contribution of spectral (400–1,000 nm) to the calibration model were shown in Figure 2. Some obvious peaks and valleys could be found at certain wavelengths which were selected as the effective wavelengths. Table 3 shows the effective wavelengths which were selected by SPA and RC with two preprocessing methods, and the wavelengths selected by SPA were ranked in the order of importance.

### 3.3. Comparison of Six Calibration Models

Four different models were developed using the selected EWs by SPA and RC. Taking the selected LVs as direct inputs, two kinds of calibration models were built. In this study, these six linear and non-linear calibration models were developed to determine the TAA in barley leaves. Table 4 shows the calibration and validation results of the six models.

Compared with the above models, the PLS models achieved acceptable results in general. The performance of the LV-PLS and LV-LS-SVM models was better than that of other models in this study, which demonstrated that latent variables included more useful information for the determination of TAA content in barley leaves. The best prediction performance was achieved by the LV-LS-SVM (Raw) model, and the correlation coefficient and RMSEP in validation were 0.937 and 0.530. Comparing with the SPA-PLS model and the PLS model with full-spectrum, for raw spectral the correlation coefficient decreased by 0.02%, but the variables decreased by 99%; for de-trending spectral the correlation coefficient increased by 0.65%, while the variables decreased by 98.84% at the same time. The results indicated that the selected wavelengths carried most useful information of full-spectral, which was important for simplifying the model and developing portable instruments. On the other hand, the effective wavelengths selected by SPA performed better than those chosen by RC in this study, probably because the effective wavelengths selected by SPA were minimally redundant.

## 4. Conclusions

PLS and LS-SVM models were successfully developed from the Vis/NIR spectra for the fast determination of total amino acid (TAA) in barley leaves. This was important as a physiological indicator in crops during plant growth and herbicide stress. Raw and de-trending methods were the optimal preprocessing methods by the PLS models. The LV-LS-SVM models with Raw spectra achieved the best prediction performance for the validation set with *r* = 0.937 and RMSEP = 0.530. The results of this study indicated that NIR spectroscopy could be used for the determination of TAA content in barley leaves. The RC and SPA methods provided helpful approaches to determine the effective wavelengths, which was useful for the development of portable instrument or sensors for plant growth monitoring. Considering the limitation of samples used in this specific study, the results indicated the feasibility of using NIR spectroscopy to detect TAA in barley leaves under herbicide stress. More leaf samples with different growth stages and barley varieties would be taken into consideration to expand and develop more stable and robust models. This study supplied a new approach for the fast and accurate detection method of physiological parameters of barley growth.

## Figures and Tables

**Table 1.** Statistics of TAA in calibration and validation sets.

**Sample Set**	**Sample No.**	**Range (mg/g DW)**	**Mean (mg/g DW)**	**Standard deviation (mg/g DW)**
**Calibration**	50	4.720–10.382	6.727	1.521
**Validation**	25	4.728–10.250	6.723	1.525
**All**	75	4.720–10.382	6.726	1.512

**Table 2.** The prediction results of TAA in validation set by the PLS models with full-spectrum.

**Pretreatment**	**LV**	***r***	**RMSEP**	**Bias**	**Slope**	**Offset**
**Raw**	6	0.879	0.751	−0.098	0.902	0.559
**SG**	6	0.868	0.790	−0.144	0.886	0.622
**SNV**	4	0.821	0.876	−0.026	0.783	1.432
**MSC**	4	0.814	0.893	−0.030	0.776	1.475
**1-Der**	6	0.823	0.866	0.031	0.769	1.582
**2-Der**	1	0.497	1.306	0.067	0.294	4.815
**De-trending**	6	0.875	0.759	−0.141	0.867	0.751
**DOSC**	1	0.835	0.909	−0.097	0.906	0.537

**Table 3.** The selected EWs by SPA and RC.

**Pretreatment**	**Methods**	**No.**	**Selected EWs/nm**
**Raw**	SPA	6	716, 976, 684, 982, 409, 407
	RC	8	409, 959, 968, 976, 982, 985, 988, 992
**De-trending**	SPA	7	747, 724, 888, 995, 415, 897, 922
	RC	11	403, 409, 897, 924, 934, 964, 968, 976, 981, 986, 989

**Table 4.** The prediction results of total amino acid (TAA) content in barley leaves by different models.

**Models**	**Pretreatment**	**LV/EW/(γ, σ^2^)**	**Calibration**		**Validation**	

			***r****_c_*	**RMSEC**	***r****_v_*	**RMSEP**
**LV-PLS**	Raw	5/-/-	0.928	0.562	0.935	0.551
	De-trending	4/-/-	0.935	0.535	0.929	0.558
**SPA-PLS**	Raw	5/6/-	0.866	0.754	0.879	0.717
	De-trending	5/7/-	0.905	0.642	0.880	0.757
**RC-PLS**	Raw	3/8/-	0.693	1.085	0.625	1.205
	De-trending	4/11/-	0.880	0.716	0.862	0.779
**LV-LS-SVM**	Raw	6/-/(68.12, 271.15)	0.935	0.540	0.937	0.530
	De-trending	6/-/(8.91 × 10^6^, 1.21 × 10^7^)	0.936	0.533	0.930	0.309
**SPA-LS-SVM**	Raw	-/6/(1.16 × 10^6^, 4.61 × 10^5^)	0.869	0.744	0.872	0.737
	De-trending	-/7/(1.11 × 10^6^, 4.74 × 10^5^)	0.906	0.638	0.877	0.776
**RC-LS-SVM**	Raw	-/8/(2.06 × 10^6^, 1.19 × 10^4^)	0.837	0.827	0.360	1.553
	De-trending	-/11/(6.66, 45.84)	0.940	0.528	0.886	0.701
